# Translating regenerative medicine therapies in neonatal necrotizing enterocolitis

**DOI:** 10.1038/s41390-024-03236-x

**Published:** 2024-05-28

**Authors:** Niloofar Ganji, Brian Kalish, Martin Offringa, Bo Li, James Anderson, Sylvain Baruchel, Martin Blakely, Paolo De Coppi, Simon Eaton, Estelle Gauda, Nigel Hall, Anna Heath, Michael H. Livingston, Carol McNair, Robert Mitchell, Ketan Patel, Petros Pechlivanoglou, Hazel Pleasants-Terashita, Erin Pryor, Milica Radisic, Prakesh S. Shah, Bernard Thébaud, Kasper Wang, Augusto Zani, Agostino Pierro

**Affiliations:** 1 Institute of Medical Sciences, University of Toronto, Toronto, ON Canada; 2 Translational Medicine, The Hospital for Sick Children Research Institute, University of Toronto, Toronto, ON Canada; 3 Program in Neuroscience and Mental Health, The Hospital for Sick Children Research Institute, University of Toronto, Toronto, ON Canada; 4 Department of Molecular Genetics, University of Toronto, Toronto, ON Canada; 5 Division of Neonatology, Department of Pediatrics, The Hospital for Sick Children, Toronto, ON Canada; 6 Child Health Evaluative Sciences, The Hospital for Sick Children Research Institute, Toronto, ON Canada; 7 Departments of Bioethics, The Hospital for Sick Children, Toronto, ON Canada; 8 Department of Surgery, University of Texas Health Science Center at Houston, Houston, TX USA; 9 Stem Cells and Regenerative Medicine, Developmental Biology and Cancer Programme, UCL Great Ormond Street Institute of Child Health, London, UK; 10 University Surgery Unit, Faculty of Medicine, University of Southampton, Southampton, UK; 11 Division of Biostatistics, Dalla Lana School of Public Health, University of Toronto, Toronto, ON Canada; 12 Department of Statistical Science, University College London, London, UK; 13 McMaster Children’s Hospital, McMaster University, Hamilton, ON Canada; 14 Neonatal Intensive Care Unit, The Hospital for Sick Children, University of Toronto, Toronto, ON Canada; 15 Micregen Ltd, Thames Valley Science Park, Reading, UK; 16 NEC Society, 140 B St. Ste 5 #128, Davis, CA USA; 17 Institute of Biomedical Engineering, University of Toronto, Toronto, ON Canada; 18 Department of Pediatrics, Mount Sinai Hospital, Toronto, ON Canada; 19 Division of Neonatology, Department of Pediatrics, Children’s Hospital of Eastern Ontario (CHEO) and CHEO Research Institute, Regenerative Medicine Program, Ottawa Hospital Research Institute, Ottawa, ON Canada; 20 Division of General and Thoracic Surgery, The Hospital for Sick Children, University of Toronto, Toronto, ON Canada; 21 Department of Physiology, University of Toronto, Toronto, ON Canada

## Introduction

Necrotizing enterocolitis (NEC) is characterized by intestinal inflammation and in severe cases, necrosis and perforation. It remains an unsolved clinical challenge with mortality rates up to 50%.^1^ NEC survivors often develop early postoperative complications, short-gut syndrome, and neurodevelopmental disabilities. There are no specific medical therapies with clinical benefit in infants with NEC. Current NEC management involves the cessation of oral feeds, decompression of the stomach with a nasogastric tube, hemodynamic support with intravenous fluids and inotropes, and administration of broad-spectrum antibiotics for gut infections.^2^ In severe disease, surgery is required to resect necrotic bowel.^2^ The high mortality and morbidity of NEC indicate the need for innovative targeted treatments.

Several risk factors are implicated in NEC, including prematurity, formula feeding, gut ischemia, genetic predisposition, and intestinal dysbiosis. The pathogenesis of NEC is multifactorial and not fully understood,^2–5^ affecting not only the intestine but also many secondary organ systems. A key aspect of NEC pathophysiology involves the disruption of intestinal epithelium by intraluminal bacteria, particularly at the intestinal villi tips.^2,4^ Endotoxins released from these bacteria bind to Toll-like receptor 4 (TLR4) on the surface of intestinal epithelial cells, activating pathogen-associated molecular pattern (PAMP) receptors, and triggering immune responses that compromise the gut barrier and enable bacterial invasion.^2–5^ This process initiates a strong inflammatory response within the gut’s lamina propria, driven by inflammatory cytokines such as tumor necrosis factor-alpha (TNF-α) and interleukin-1β (IL-1β). Furthermore, the activation of TLR4 on endothelial cells impairs intestinal perfusion, leading to mesenteric ischemia and enterocyte injury.^2,6^ Additionally, TLR4 activation hampers intestinal repair processes by inhibiting enterocyte proliferation and migration.^4^ Vasoactive substances such as platelet-activating factor (PAF), endothelin-1 (ET-1), and nitric oxide (NO) are also released in response to inflammation, further exacerbating intestinal damage by impairing blood flow and vasorelaxation in the intestinal microvasculature.^7^ Importantly, NEC is characterized by depletion of intestinal stem cells, linked to defective Wnt signaling,^8,9^ a key pathway in stem cell regulation and homeostasis.

Preclinical studies have made important discoveries in the use of stem cells,^10–14^ and their products such as secretomes (conditioned medium),^15^ or extracellular vesicles,^8,14,16,17^ to regenerate the injured intestine in NEC. Stem cell-based interventions have shown promise in mitigating the multifaceted pathogenesis of NEC by enhancing anti-inflammatory, anti-apoptotic, and regenerative processes. Stem cells and their products exert their beneficial effect in NEC by inhibiting TLR4 and other inflammatory pathways,^10,18^ preserving gut barrier function,^12,13,19^ promoting epithelial regeneration through paracrine mechanisms,^8,10,20^ and reducing tissue injury and enterocyte apoptosis.^10,21^ Reduced NEC incidence and severity and improved survival are among the most important outcomes achieved in preclinical studies. Importantly, stem cell-based interventions have demonstrated benefit both for NEC prevention,^19,21,22^ as well as rehabilitation post-NEC.^23^ These interventions not only address the underlying inflammatory and apoptotic pathways but also support the repair and renewal of the intestinal epithelium, crucial for restoring normal function. Hence, a regenerative medicine approach for NEC treatment could potentially avoid long-term complications of NEC such as development of short-gut syndrome – a severe consequence of extensive intestinal resection in NEC and improve neurodevelopmental outcomes by mitigating the impact of NEC on the developing brain. Hence, stem cell-based therapies hold great potential in the clinical management of NEC.

In related neonatal conditions, phase I clinical trials conducted in extremely low birth weight neonates with bronchopulmonary dysplasia (BPD),^24,25^ hypoxic-ischemic encephalopathy (HIE),^26^ or severe intraventricular hemorrhage (IVH)^27^ have demonstrated that stem cell-based interventions are feasible. However, other than in one case report,^28^ stem cell-based interventions have not yet been evaluated in neonates with NEC. This gap presents a significant opportunity to advance NEC treatment, fueling an urgent need for confirmatory studies and studies to test the safety and feasibility of these interventions in infants with NEC. Yet, the clinical translation of stem cell interventions for NEC faces several challenges,^29^ including lack of a streamlined clinical trial framework, limited interdisciplinary collaboration, and minimal parent/caregiver engagement.

## Lessons learned from other fields

The success of chimeric antigen receptor (CAR) T-cell therapies for certain cancers is a perfect example of precision translational medicine being used to treat a refractory medical condition.^30^ Since 2017, six CAR T-cell therapies have been approved by the Food and Drug Administration. The success of CAR T-cell therapy can be attributed to robust trial designs following a stepwise approach, impressive response rates, successful management of therapy-related side effects, and successful industry partnerships. Another example of a successful regenerative medicine effort in neonates is the INCuBAToR project (Innovative Neonatal Cellular Therapy for Bronchopulmonary Dysplasia (BPD): Accelerating Translation of Research).^31^ This project has brought together multidisciplinary partners to safely introduce stem cell therapy for BPD. The INCuBaToR facilitates sharing of knowledge and resources to optimize trial design, patient recruitment, and regulatory compliance, highlighting the value of a collaborative platform in accelerating the clinical translation of stem cell-based therapies for neonatal diseases.

Inspired by these efforts, and in response to the pressing need for innovative therapies for NEC, we recently organized a convergent working group (CWG) meeting funded by the University of Toronto’s Medicine by Design program, comprising a multidisciplinary team of experts to discuss existing knowledge gaps and steps needed to successfully translate stem cell-based therapy into clinical evaluation. With the participants’ consensus, we have synthesized the key conclusions of the CWG meeting into a novel approach: the NEC-ACCELERATOR (NECrotizing enterocolitis-AC*celeration of* CELl-basEd *&* R*egenerative medicine* A*dvancements for* T*ranslation in ne*O*nates)*, a multidisciplinary approach to enhance the success of ethical, safe, and timely clinical translation.

In this *special article*, we *(i)* present the NEC-ACCELERATOR as an engine for development and execution of neonatal regenerative medicine trials, and *(ii)* outline strategic priorities for translating stem cell-based therapies in NEC.

## Nec-accelerator: enhancing successful clinical translation of stem cell-based interventions for NEC treatment

**Fig. 1:**
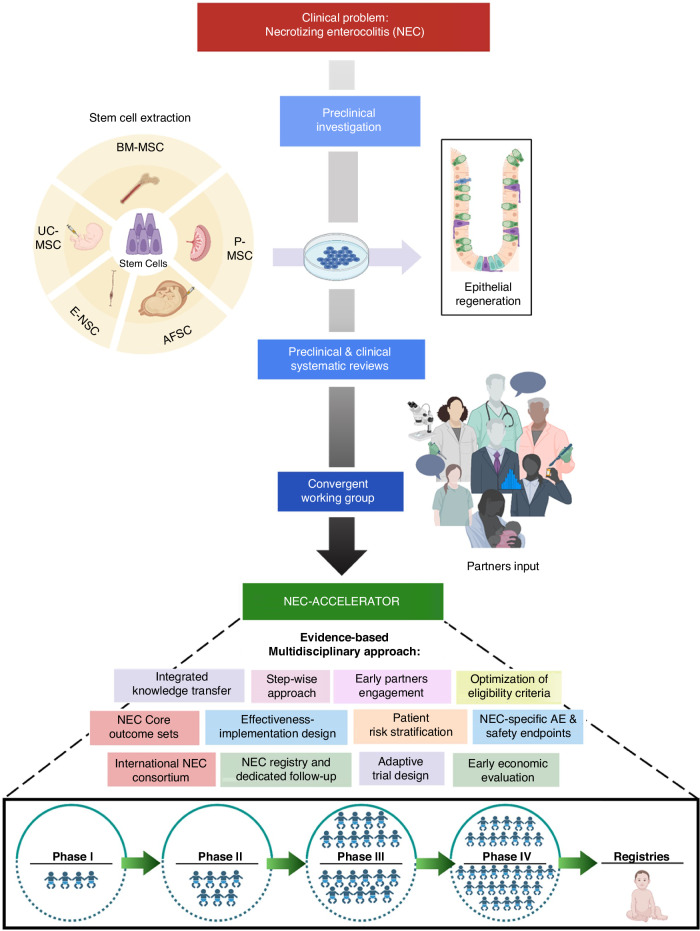
NEC-ACCELERATOR uses an evidence-based multidisciplinary approach to enhance the success of safe and timely clinical translation of stem cell-based therapies for neonates with NEC. BM-MSC bone marrow-derived mesenchymal stem cells, UC-MSC umbilical cord-derived MSC, E-NSC enteric neural stem cells, AFSC amniotic fluid-derived stem cells, P-MSC placenta-derived MSC.

The NEC-ACCELERATOR is a multidisciplinary initiative for streamlining the design and coordination of clinical trials for neonates with NEC (Fig. 1). This initiative serves as a *prototype* for future neonatal regenerative medicine trials across Canada and internationally. The NEC-ACCELERATOR is designed to identify and overcome barriers to translation in an efficient and structured manner, and to establish consensus on the definition, characterization, and guidelines for manufacturing and use of stem cell-based interventions in NEC.

The NEC-ACCELERATOR harmonizes the diverse expertise of key trial Partners, a multidisciplinary group composed of experts in various fields and trial stakeholders (Table 1). The NEC-ACCELERATOR Partners include parents/caregivers, nurses, neonatologists, pediatric surgeons, scientists specializing in stem cell biology, intestinal epithelial biology, and immunology, trialists, biostatisticians, experts in research ethics and regulatory compliance, health economists, industry partners, implementation scientists, and professionals in broadcasting and communications. This comprehensive collaboration is further enriched by partnerships with patient-centered organizations such as the Canadian Neonatal Network (CNN) and NEC Society (www.necsociety.org), ensuring that the initiative is deeply rooted in the needs and experiences of those most affected by NEC.

**Table. 1:** NEC-ACCELERATOR partners.

	Discipline/Body	Reasons for inclusion
1	Parents/Caregivers	To incorporate the perspective, concerns, and preferences of those directly affected, from clinical trial design to implementation of study results
2	Nursing	To ensure proper care, communication, and patient management during trials
3	Basic Science (stem cell biology, intestinal epithelial biology, and immunology)	To provide foundational knowledge and research expertise in NEC and stem cell therapies
4	Clinicians (neonatologist and pediatric surgeon)	To offer specialized knowledge in neonatal care and management of NEC and potential integration of stem cell therapies
5	Trials methods	To design efficient, adaptive clinical trials that generate the evidence for safety and efficacy needed to inform treatment decisions
6	Biostatistics	To provide statistical analysis and design support for trials
7	Research ethics	To address ethical challenges and considerations in neonatal clinical trials
8	Regulatory compliance	To navigate regulatory processes, guidelines, and requirements
9	Health economics	To evaluate financial viability and inform decision-making related to stem cell therapies
10	Industry partner (Micregen Ltd)	To collaborate in the development of standardized stem cell products and manufacturing processes
11	Implementation science	To facilitate effective adoption and integration of stem cell therapies in NEC management
12	Broadcasting and communication	To aid in disseminating information, raising awareness, and fostering communication among Partners
13	Patient-centered organizations (Canadian Neonatal Network and NEC Society)	To offer “lived experience” insights and trauma-informed care practices, advocate, and support research and translation of therapies

The core objectives of the NEC-ACCELERATOR include: *(i)* conducting systematic reviews of preclinical and clinical studies that provide an evidence-based summary of current knowledge and set the research agenda for clinical translation, *(ii)* facilitating integrated knowledge exchange through early engagement of key Partners, *(iii)* developing consensus-based NEC diagnosis and eligibility criteria, and safety and clinical outcomes most relevant to the intervention, *(iv)* determining the optimal intervention mode, dose, and timing in the disease course to proceed into clinical evaluation for NEC, *(v)* conducting early economic evaluation of new therapies, and *(vi)* launching prospective cohort studies to define eligibility criteria in future trials.

The implementation strategy of the NEC-ACCELERATOR includes the initial setup of prospective observational cohort studies targeting stem cell interventions in NEC. These will pave the way for the subsequent launch of phase I safety and phase I/II safety/feasibility trials, ultimately streamlining the transition to phase II and phase II/III efficacy and effectiveness trials. By integrating the diverse expertise of its Partners, and leveraging early and comprehensive engagement of Partners, the NEC-ACCELERATOR aims to catalyze the advancement of stem cell interventions for NEC treatment. Through this initiative, we aim to not only advance the scientific understanding and treatment options for NEC but also to create a template that can be adapted and applied to other neonatal conditions, ultimately enhancing care and outcomes for our most vulnerable patients worldwide.

## Strategic priorities for successful translation of stem cell therapies for NEC (Fig. 1)

### Empowering neonatal intensive care unit (NICU) teams and enhancing parent/caregiver engagement

The success of regenerative medicine trials for NEC hinges significantly on the empowerment of NICU teams and the active engagement of parents and caregivers.^29^ Comprehensive training for NICU team members that focuses on the informed consent process and includes a trauma-informed approach to addressing concerns of parents/caregivers is pivotal for trial success. Effective training strategies ensure that parents/caregivers are adequately informed and supported, thereby facilitating their engagement and mitigating follow-up losses. Many parents/caregivers learn about NEC after diagnosis. The overwhelming psychological burden may hinder their understanding of intervention benefits/risks, and active participation in the trial. Providing clear, accessible information about NEC, including risk factors and proposed interventions before diagnosis can foster informed decision-making. Technological tools (e.g. animated videos) can help to inform parents/caregivers about regenerative medicine interventions and trial procedures.^31^ We have partnered with the NEC Society, the only patient-led nonprofit organization working to advance research, advocacy, and education to improve treatment strategies and outcomes for the most vulnerable infants at risk for NEC. The perspectives and lived experiences of parents/caregivers affected by NEC significantly contribute to translational research trial design and improve consent processes.

### Stepwise approach to overcome recruitment challenges

Building upon the foundation of empowered NICU teams and engaged parents/caregivers, the next logical step is to address the recruitment challenges that frequently impede the progress of pediatric and neonatal trials, especially in NEC.^32,33^ Slow recruitment is a critical issue that can lead to early discontinuation of pediatric and neonatal trials. This is exacerbated by complicating factors such as rarity of the disease, restrictive eligibility criteria, concerns about stem cell interventions, inadequate risk/benefit assessment, limited partner engagement, and lack of institutional support.^31^ These factors are especially pertinent in NEC, an acute, rare, life-threatening condition affecting the most vulnerable patients. To navigate these challenges, a *stepwise* and *evidence-based approach* is essential. This approach relies on empirical evidence gathered at each of the steps below, which inform and refine the subsequent step(s):

(i) Designing eligibility criteria, adverse events, and outcomes based on historical cohort studies such as the Canadian Neonatal Network^34^;
(ii) Conducting observational studies and trial feasibility studies to refine eligibility criteria, pre-specified adverse events, pilot patient and patient-family inclusion strategies, intervention delivery, and outcome measurements;
(iii) Developing protocols for phase I/II safety and feasibility interventional trials;
(iv) Developing protocols for phase II/III efficacy trials, which will evaluate the effectiveness of interventions.

Careful planning, resource allocation, and a methodological approach informed by empirical evidence is the key to addressing recruitment challenges, allowing advancement into the critical phases of trial development.

### Core outcome sets and adaptive trial design

The large heterogeneity in outcome selection, measurement, and reporting in NEC trials hinders successful interpretation and implementation of findings. Following the Standard Protocol Items: Recommendations for Interventional Trials (SPIRIT) Guidelines for Reporting Outcomes in Trial Protocols,^35^ outcomes should address five core elements: outcome domain (title or concept), measurement variable and tool, specific metric (unit of measurement), method of aggregation (procedure for estimating treatment effect), and time point of outcome data collection. A harmonized core outcome set for NEC treatment trials involving stem cell-based interventions considering the most relevant outcomes for clinicians, patients, and parents/caregivers is required to guide the development of evidence-based regenerative medicine interventions.

Early involvement of trial statisticians using modern frameworks can address challenges related to small sample sizes and heterogeneous patient populations, minimizing recruitment time and improving trial efficiency through adaptive trial designs and interim data analysis. Adaptive trial designs allow for preplanned modifications to the trial based on interim data analyses, including changes to sample size, treatment allocation, and even the inclusion/exclusion criteria. Techniques like Bayesian statistics^36^ and response-adaptive randomization^37^ incorporate prior and accumulating knowledge to adjust trial parameters dynamically, reducing the time required to get effective, safe combination therapies to the most vulnerable patients. Furthermore, adaptive trial designs offer a strategic advantage with the potential for substantial cost savings. The high cost of running trials, driven by the extensive time and resources needed for trial activation and to meet accrual targets, poses a significant barrier to their long-term feasibility and impacts the scope and scale of clinical research undertaken.^38^ The rarity of NEC necessitates the inclusion of multicenter trials to gather sufficient number of participants, further escalating costs. Moreover, the vulnerability of the patient population, predominantly premature infants, adds layers of complexity in trial design and implementation, requiring rigorous safety protocols and extended monitoring periods. Adaptive designs can mitigate these financial challenges by reducing unnecessary expenditure on ineffective treatments or overly large sample sizes.

### Hybrid effectiveness-implementation design

The transition from methodologically robust trial designs to the clinical implementation of new treatments, once proven effective through high quality clinical trials, is often delayed or never occurs. This is due to various factors including intervention complexity. Important determinants of implementation include attitudes of medical staff and parents/caregivers towards stem cell-based interventions, and the impact of evidence from evaluative studies on decision-making. A hybrid effectiveness-implementation design could address the delay in implementation by assessing the intervention safety and effectiveness while also exploring implementation barriers.^39^ Empirically investigating these factors during the safety/efficacy trial phase, especially understanding parent attitudes towards interventions, could be a novel approach.

### Multicenter involvement and patient risk stratification

A multinational consortium is essential for incorporating diverse NEC patients and management practices and evaluating stem cell-based interventions. Our international NEC consortium involving 12 centers across Canada, US, UK, Netherlands, Sweden, and Spain,^40^ unifies resources, expertise, input from Partners, and patient populations for effective large-scale trial design, conduct, and analysis. The consortium standardizes trial procedures, raises NEC awareness, and advocates for policy and public support, fostering advancement in stem cell-based NEC interventions. Additionally, collaboration with the Neonatal Research Network (NRN), a network of neonatal intensive care units across the United States, will further enhance the capacity for rigorous patient evaluation. Leveraging the NRN’s infrastructure and extensive experience in multi-center neonatal trials, including in NEC,^41^ in combination with the strength of our consortium, will accelerate the development and testing of innovative interventions.

In trials involving small, heterogeneous, and critically ill groups like neonates with NEC, assessing the generalizability of stem cell-based interventions is challenging. Innovative methods like risk and effect score analyses^42^ can address this by grouping patients based on predicted health outcomes or treatment effects, offering *personalized* treatment opportunities.^43^ This novel approach to treatment effect heterogeneity demands multidisciplinary expertise for effective implementation. This nuanced approach underscores the necessity for multidisciplinary expertise and collaboration in overcoming the inherent challenges of neonatal clinical trials, particularly in NEC.

### NEC-specific safety endpoints and ethical considerations

A main task of the dedicated research consortium is the development of NEC-specific adverse event (AE) frameworks and safety endpoints across collaborating centers. These frameworks will categorize AEs by type and severity and link them to stem cell-based interventions, ensuring patient safety, accurate reporting, and effective safety monitoring. Instruments for grading of unexpected AEs^44^ are essential for quantifying the severity and impact of AEs with enhanced objectivity. A Delphi consensus approach can be employed to develop a comprehensive harmonized AE framework. The Delphi method is a structured communication technique, involving the systematic collection of expert opinions.^45^ This process involves iterative rounds of questionnaires sent to a panel of selected experts and patient-families. Feedback is aggregated and shared with the panel after each round, allowing for the refinement of opinions and convergence towards a consensus. By gathering insights from key trial Partners (Table 1), including parents/caregivers, the Delphi method facilitates the creation of a harmonized AE framework most relevant to neonates with NEC in stem cell trials, enhancing the safety, efficacy, and transparency of interventions in this vulnerable population. Developing these frameworks in the early planning stages of phase I safety trials will allow for consistent safety assessment of these interventions in various trials and settings.

Following the establishment of frameworks to evaluate the safety of interventions, it is crucial to recognize that conducting neonatal stem cell-based intervention trials embodies significant ethical challenges, including minimizing risks and maximizing the value of knowledge gained. Key to both is rigorous preclinical evidence assessment and implementation of a robust consent process to avoid therapeutic misconception and misestimation by the parents/caregivers of these vulnerable, critically ill newborns.

### Industry partners and health economics

Industry partners are crucial in selecting standardized, clinical-grade stem cell products for NEC treatment. They facilitate the evaluation of product quality standards, scale, costs, labeling, packaging, storage, timely distribution, and regulatory compliance. Furthermore, early involvement of industry partners facilitates the navigation of regulatory compliance with Good Manufacturing Practices.

Early health economic evaluation, which is underutilized in neonatology, is a tool to support product investment decision making, provide guidance in (economic) data collection and support prioritization of future research. Estimation of the economic burden of NEC, as the first step, could highlight its societal and healthcare impact, and guide policy makers in prioritizing treatments. Early engagement of health economics experts to ascertain financial viability is crucial to identify whether and under which conditions, once proven effective, stem cell-based therapies for NEC are economically viable and accessible.

## Summary

To date, neonates with NEC have not benefited from novel therapeutic trials due to the key challenges in clinical translation of scientific discoveries in this population, as discussed above. Through a collaborative approach and fostering a diverse range of expertise, the NEC-ACCELERATOR aims to identify and navigate the barriers to regenerative medicine trials for this patient population. The future steps of the NEC-ACCELERATOR are summarized below.

The NEC-ACCELERATOR will devise strategies to engage clinicians, nurses, multidisciplinary members within the NICU and parents/caregivers in the process of high-quality trial design and conduct. These strategies will include providing training and resources for NICUs at our international collaborating centers, specifically tailored for regenerative medicine trials in neonatology.

The need for reliable diagnostic criteria and criteria for the design and conduct of clinical trials with consistent and clinically meaningful outcome measures are among the key hurdles impeding the development and regulatory approval of interventions for NEC treatment.^46^ The establishment of the International Neonatal Consortium (INC) by the Critical Path Institute and the Food and Drug Administration in 2015, specifically focusing on necrotizing enterocolitis (NEC), has highlighted these needs as critical steps forward.^47^ Making an early and adequate diagnosis of NEC remains one of the primary challenges in neonatology due to rapid onset and variability in clinical presentation of NEC, lack of a reliable and specific biomarker or pathognomonic sign, and shortcomings in the current diagnostic criteria used for NEC. Previous attempts have been made to develop standardized criteria for NEC diagnosis.^40,47,48^ Building upon this previous work, the NEC-ACCELERATOR aims to employ a Delphi method to further refine these criteria, as well as the eligibility criteria for infants with NEC in stem cell trials. Additionally, building upon current work to develop an international core outcome set for NEC,^49^ we aim to achieve consensus of key trial Partners on the most appropriate safety and clinical outcomes to be evaluated and reported in stem cell trials in NEC. These efforts will pave the way for more effective and timely regulatory approval of interventions and inform a comprehensive and standardized framework that supports all phases of trial development and implementation.

The establishment of a dedicated international consortium represents a significant step forward in our multicenter involvement. This consortium will promote early involvement of trial Partners in the design and conduct of initial stem cell trials in NEC worldwide. Moreover, bringing together experts in stem cell biology, intestinal epithelial biology, and immunology, in collaboration with industry partners, regulatory bodies, and health economic experts, we will determine the precise mode, dose, and timing of stem cell interventions for clinical evaluation in infants with NEC.

Through the NEC-ACCELERATOR, the initial prospective observational cohort studies for regenerative medicine therapy in NEC, followed by phase I safety and phase I/II safety/feasibility trials will be launched. By systematically addressing the challenges and barriers in translating stem cell-based therapies for neonates with NEC, the NEC-ACCELERATOR will ensure that innovative regenerative medicine therapies reach the bedside. This strategy not only promises to accelerate the availability of new treatments for NEC but also sets a precedent for future therapeutic development in neonatal care.
